# Quantitative Assessment of the Effects of Reducing Agents on Biological Macromolecules and on the Possible Repair of Oxidative Damage

**DOI:** 10.1155/2018/5704016

**Published:** 2018-08-02

**Authors:** Jianan Zhao, Naijin Xu, Hui Liu

**Affiliations:** ^1^College of Medical Laboratory, Dalian Medical University, Dalian 116044, China; ^2^Department of Chemistry, Dalian Medical University, Dalian 116044, China

## Abstract

**Objective:**

To quantitatively assess the influence of reducing agents on biological macromolecules and on the possible repair of oxidative damage.

**Methods:**

Samples (antibody, enzyme, DNA, and diluted serum) were treated with reducing agents (ammonium ferrous sulfate, ascorbic acid, potassium iodide, and sodium hyposulfite) in the experimental group and with NaCl in the control group. Enzyme-linked immunosorbent assay and quantitative PCR were used to determine the activity of antibody, enzyme, and DNA. Native gel electrophoresis (Native-PAGE) and sodium dodecyl sulfate polyacrylamide gel electrophoresis (SDS-PAGE) were used to determine protein structure. Reducing agents that had no inhibitory effect on biological macromolecules were selected. Antibodies were treated with oxidants to caused oxidative damage and then treated with reducing agents, and the possible repair of oxidative damage was assessed.

**Results:**

Certain concentrations of ammonium ferrous sulfate resulted in significant inhibition of antibody, enzyme, DNA, and diluted serum. Certain concentrations of ascorbic acid resulted in significant inhibition of antibody. Sodium hyposulfite and potassium iodide had no effect on antibody, enzyme, DNA, and diluted serum. The OD values in group A (in which HBsAb was treated by oxidation and then a reductant) were significantly higher than those in group B (HBsAb treated by oxidation).

**Conclusion:**

Ammonium ferrous sulfate, ascorbic acid, sodium hyposulfite, and potassium iodide had different effects on antibody, enzyme, DNA, and diluted serum. The reduction in antibody activity due to an oxidant was partially repaired by a reductant.

## 1. Introduction 

Oxidative stress is an imbalance between oxidation and antioxidation in the body, which tends to oxidation [1–3]. Negative effects are produced in the body by oxidizing substances, and this is thought to be an important factor in aging and disease [2, 4, 5]. The structures of various biological macromolecules, such as proteins, lipids, carbohydrates, and DNA, are altered by oxidation [3, 5–7]. Modification of amino acid residues is the main cause of protein damage and may result in breakage, hydrolysis, peptide crosslinking, and polymerization, which lead to changes in the structure and conformation, thereby affecting the biological activity of the protein [1, 6].

Antioxidation is the process of slowing down or preventing oxidation [3, 8, 9]. Substances that interfere with the initiation and diffusion of free radical chain reactions or inhibit the reactions of free radicals are called antioxidants. Antioxidants are usually reductants. Antioxidants are thought to counteract the effects of oxidants and reduce oxidative damage to the body [3, 5]. However, research on the possible repair of oxidative damage by reducing agents has rarely been reported. This study attempts to investigate the possible repair of oxidative damage in proteins from the viewpoint of chemistry.

Many researchers believe that high concentrations of antioxidants in tissues are good for the body, but some researchers are skeptical [5, 10, 11]. Some studies have found that there are some chemical side effects of reducing agents [10, 12, 13]. Therefore, to investigate the repair of oxidative damage, reducing agents with no inhibitory effects on bioactive molecules should be selected first. In this study, samples (antibody, enzyme, DNA, and diluted serum) were treated with well-known reducing agents (ammonium ferrous sulfate, ascorbic acid, sodium hyposulfite, and potassium iodide) in the experimental group and with NaCl in the control group. The reducing agents (sodium hyposulfite and potassium iodide) that had no inhibitory effect on biological macromolecules were selected with using enzyme-linked immunosorbent assay (ELISA) and quantitative PCR (qPCR) to determine the activity of antibody, enzyme, and DNA. Antibodies were treated with oxidants to cause oxidative damage and then treated with reducing agents, and the possible repair of oxidative damage was assessed. We believe that biological activity can be recovered following oxidative damage using safe reducing agents, which is the experimental foundation for antioxidant treatments* in vitro* and/or* in vivo*.

## 2. Materials and Methods

### 2.1. Effects of Reducing Agents on Antibody Activity

*2.1.1.* Reducing agents (6 mmol/L ammonium ferrous sulfate, 6 mmol/L ascorbic acid, 60 mmol/L sodium hyposulfite, and 60 mmol/L potassium iodide) were diluted at a 1:1 ratio using distilled water to obtain eight concentrations. NaCl solutions were prepared with the same pH and ionic strength as the reductant solvent.

*2.1.2.* The samples (HBsAb, healthy human mixed serum with an antibody to hepatitis B surface antigen, collected from a hospital in Dalian, China) were mixed with a reductant solution or NaCl solution in a test tube at a ratio of 1:5 at 4°C for 1 h.

*2.1.3.* The antibody activity was detected with ELISA (hepatitis B virus surface antibody test kit, Chen Yu, China). The OD values were used to assess antibody activity, with higher OD values indicating higher antibody activity. The OD value for the blank was 0.042, which is eliminated in the following calculation. Inhibition rate = [(the OD value of the control group – the OD value of the blank hole) – (the OD value of the experimental group – the OD value of the blank hole)]/(the OD value of the control group – the OD value of the blank hole)×100%. Data were analyzed using GraphPad Prism 5 and are presented as the mean ± SD. Statistical significance was determined using Student's *t*-test and is presented as ^*∗*^(*p* < 0.05), ^*∗∗*^(*p* < 0.01), or ^*∗∗∗*^(*p* < 0.001).

### 2.2. Effects of the Reducing Agents on Protein Structures

*2.2.1.* Reducing agents were prepared at the following concentrations: 7.8 mmol/L ammonium ferrous sulfate, 7.8 mmol/L ascorbic acid, 78 mmol/L sodium hyposulfite and 78 mmol/L potassium iodide, and NaCl solutions were prepared at the concentrations of 7.8 and 78 mmol/L.

*2.2.2.* The diluted serum (healthy human serum, collected from a hospital in Dalian, China) was mixed with a reductant solution or NaCl solution in a test tube at a ratio of 1:4 at 4°C for 1 h and protected from light.

*2.2.3.* Native-PAGE and SDS-PAGE were used to investigate whether the spatial structures and molecular structures of the proteins had changed [14–16]. Separation gels and concentrated gels were established at 12% and 5%. Concentrated gels were electrophoresed for 35 min; separation gels were electrophoresed for 80 min, followed by staining with Coomassie Brilliant Blue, and then decolorized.

### 2.3. Effects of the Reducing Agents on the Activity of Taq Enzyme and DNA

*2.3.1.* Reducing agents (10 mmol/L ammonium ferrous sulfate, 10 mmol/L ascorbic acid, 100 mmol/L sodium hyposulfite, and 100 mmol/L potassium iodide) were diluted at a 1:1 ratio using distilled water to obtain eight concentrations. NaCl solutions were prepared with the same pH and ionic strength as the reductant solvent. The Taq enzyme (SYBR® Premix Ex Taq TMII, Tli RNaseH Plus, Takara, Japan) was mixed with a reductant solution or NaCl solution in a test tube at a ratio of 1:1 and the solutions were placed for 1 h and protected from light. Other ingredients were not treated except enzymes.

*2.3.2.* Reducing agents (6 mmol/L ammonium ferrous sulfate, 6 mmol/L ascorbic acid, 60 mmol/L sodium hyposulfite, and 60 mmol/L potassium iodide) were diluted at a 1:1 ratio using distilled water to obtain eight concentrations. NaCl solutions were prepared with the same pH and ionic strength as the reductant solvent. DNA samples were extracted using a DNA extraction kit (Omega Biotek, Norcross, GA, USA). The primers for genes used in this study were ß-actin (F:5′-CAAATATGAGATGCGTTGTTCAGG-3′, R:5′-TGTGTGGACTTGGGAGAGGA-3′) and CRP (F:5′-AATGTGAACATGTGGGACTTTGTG-3′, R:5′-CGCCAGTTCAGGACATTAGGAC-3′). The DNA was mixed with a reductant solution or NaCl solution in a test tube at a ratio of 1:5, and the solutions were placed for 1 h and protected from light. Other ingredients were not treated except DNA.

*2.3.3.* Ct values were determined using qPCR, and corresponding Ct values for the same gene in the experimental group and the control group were recorded; Inhibition rates were calculated using the following equation: Inhibition rate = (Ct value of the experimental group – Ct value of the control group)/Ct value of the control group ×100%. Data were analyzed using GraphPad Prism 5 and are presented as the mean ± SD. Statistical significance was determined using Student's *t*-test and is presented as ^*∗*^(*p* < 0.05), ^*∗∗*^(*p* < 0.01), or ^*∗∗∗*^(*p* < 0.001).

### 2.4. Possible Reversion of Oxidative Damage

**Figure 1:**
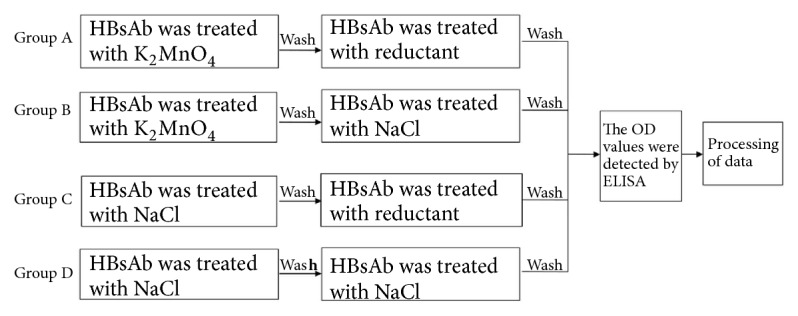
The grouping method and sample processing methods.

*2.4.1.* Reductants with no significant inhibitory effects on the activity of the antibody, enzyme, DNA,and protein structures were selected via the procedures in Sections 2.1, 2.2, and 2.3. The antibody to HBsAg was treated with potassium hypermanganate (25 mmol/L) to cause oxidative damage [17]. After removing the oxidant, the antibody was treated with reductant (50 mmol/L sodium hyposulfite and 50 mmol/L potassium iodide; the experimental concentration was obtained from a previous experiment). The experiment included four groups: group A, group B, group C, and group D. The grouping method and sample processing methods in each group are shown in Figure 1.

*2.4.2.* Hepatitis B surface antigen samples (recombinant hepatitis B vaccine, Han Xin Biopharmaceutical Company, Dalian, China) (1:100 dilution) were added to the plate, and the OD values were determined with ELISA (hepatitis B virus surface antigen test kit, Chen Yu, China). The OD values in group A, group B, group C, and group D were recorded. Data were analyzed using GraphPad Prism 5. Statistical significance was determined using Student's *t*-test and is presented as the mean ± SD and *P* value.

## 3. Results

### 3.1. The Effects of Reductants on Antibody

The inhibitory rate in the experimental group was significantly different to that in the control group when the concentration of ammonium ferrous sulfate was 5, 2.5, 1.25, 0.625, and 0.3125 mmol/L. The inhibitory rate in the experimental group was not significantly different to that in the control group when the concentration of ammonium ferrous sulfate was 0.15625, 0.078125, and 0.0390625 mmol/L (Table 1). The inhibitory rate in the experimental group was significantly different to that in the control group when the ascorbic acid concentration was 5, 2.5, 1.25, and 0.625 mmol/L. The inhibitory rate in the experimental group was not significantly different to that in the control group when the ascorbic acid concentration was 0.3125, 0.15625, 0.078125, and 0.0390625 mmol/L (Table 1). The inhibitory rate in the experimental group was not significantly different to that in the control group when the concentration of potassium iodide and sodium hyposulfite were 50, 25, 12.5, 6.25, 3.125, 1.5625, 0.78125, and 0.390625 mmol/L (Table 2).

### 3.2. The Effects of Reductants on the Structure of Serum Proteins

**Figure 2:**
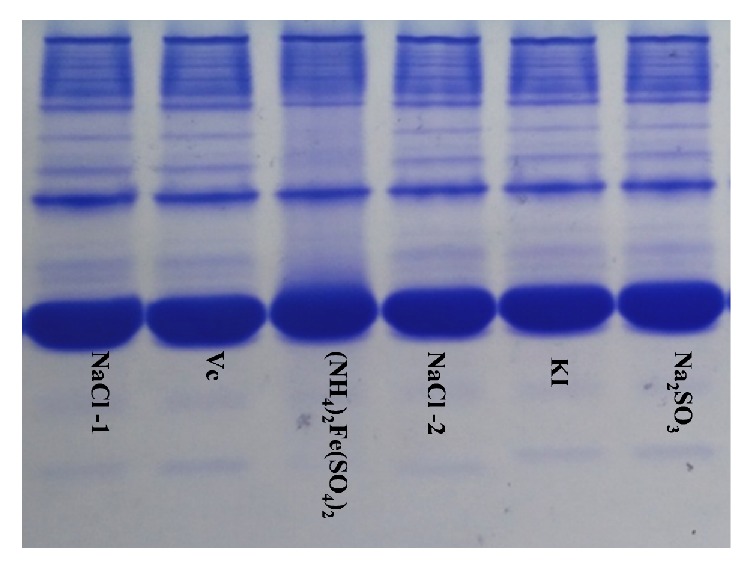
The effects of reductants on the spatial structure of proteins—Native-PAGE.

**Figure 3:**
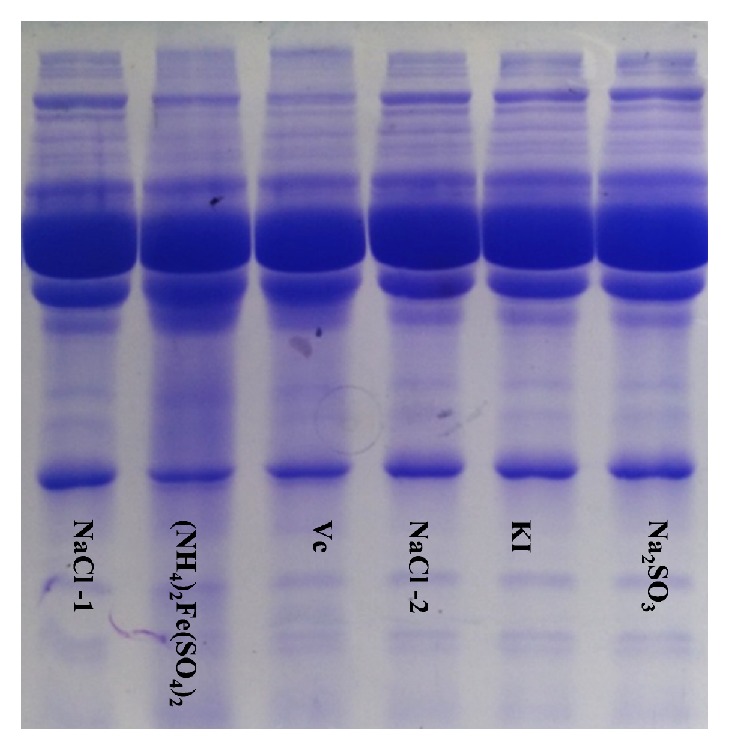
The effects of reductants on protein molecular structures—SDS-PAGE.

The serum protein bands treated with ammonium ferrous sulfate were deficient and fuzzy compared with those treated with NaCl. The serum proteins treated with ascorbic acid, potassium iodide, and sodium hyposulfite were unchanged compared with those treated with NaCl (Figures 2 and 3).

### 3.3. The Effects of Reductants on Taq Enzyme

The inhibitory rate in the experimental group was significantly different to that in the control group when the concentration of ammonium ferrous sulfate was 5, 2.5, 1.25, and 0.625 mmol/L (Table 3). The inhibitory rate in the experimental group was not significantly different to that in the control group when the concentration of ammonium ferrous sulfate was 0.3125, 0.15625, 0.078125, and 0.0390625 mmol/L (Table 3). The inhibitory rate in the experimental group was not significantly different to that in the control group when the ascorbic acid concentration was 5, 2.5, 1.25, 0.625, 0.3125, 0.15625, 0.078125, and 0.0390625 mmol/L (Table 3). The inhibitory rate in the experimental group was not significantly different to that in the control group when the concentrations of potassium iodide and sodium hyposulfite were 50, 25, 12.5, 6.25, 3.125, 1.5625, 0.78125, and 0.390625 mmol/L (Table 4).

### 3.4. The Effects of Reductants on DNA

The inhibitory rate in the experimental group was significantly different to that in the control group when the concentration of ammonium ferrous sulfate was 5, 2.5, 1.25, 0.625, 0.3125, 0.15625, 0.078125, and 0.0390625 mmol/L (Table 5). The inhibitory rate in the experimental group was not significantly different to that in the control group when the ascorbic acid concentration was 5, 2.5, 1.25, 0.625, 0.3125, 0.15625, 0.078125, and 0.0390625 mmol/L (Table 5). The inhibitory rate in the experimental group was not significantly different to that in the control group when the concentrations of potassium iodide and sodium hyposulfite were 50, 25, 12.5, 6.25, 3.125, 1.5625, 0.78125, and 0.390625 mmol/L (Table 6).

### 3.5. The Possible Repair of Oxidative Damage

The results of Sections 3.1, 3.2, 3.3, and 3.4 were comprehensively analyzed, and the reagents (50 mmol/L sodium hyposulfite and 50 mmol/L potassium iodide; the experimental concentration was obtained from a previous experiment) that had no effect on the antibody, enzyme, DNA, and protein structures were selected. In this experiment, the OD values in group A (HBsAb was treated by oxidation and then a reductant) were significantly higher than those in group B (HBsAb treated by oxidation). The OD values in group B were significantly lower than those in group D (HBsAb not treated with a reductant or oxidant). The OD values in group C (HBsAb treated with a reductant) were not significantly different to those in group D. These results are shown at Tables 7 and 8.

## 4. Discussion

This study comprehensively evaluated the effects of several reducing agents on the activity of biological macromolecules. On this basis, whether the damage to biological macromolecules caused by oxidation can be repaired by a reducing agent was investigated. Firstly, ELISA was used to analyze the effect of each reductant on antibody activity. Hepatitis B surface antibody test kit was used in this experiment, and the optimal concentrations of reagents and diluted concentrations of samples were obtained through pre-experiments. Because an antibody is an active protein, temperature, pH, ionic strength, and so on have certain influences on its activity [18, 19]. The ionic strength and pH values of the control solutions were in each case matched to those of the appropriate experimental group by calculation and adjustment, and samples of the experimental group and corresponding control group were added to the same plate at the same temperature. The effects of pH, ionic strength, and temperature on the experimental results were thus eliminated, and the experimental results are only related to the actions of the reducing agents. The results showed that ammonium ferrous sulfate had an inhibitory effect on the activity of antibodies at concentrations of 5 mmol/L, 2.5, 1.25 0.625, and 0.3125 mmol/L, ascorbic acid had an inhibitory effect on the activity of antibodies at concentrations of 5, 2.5, 1.25, and 0.625 mmol/L. potassium iodide and sodium hyposulfite had no inhibitory effects on the activity of antibodies in the concentration range used in the experiment.

Secondly, native-PAGE and SDS-PAGE were used to compare the protein bands of the experimental group and control group on the same gel and to explore whether the spatial structures and molecular structures of the proteins had changed. There were no reductive substances in the gel or buffer for native-PAGE, and each sample was directly spotted for electrophoresis without heating or degeneration, so that the proteins maintained their natural biological activity and were separated according to their natural shapes and charges [14, 15]. The results showed that the protein bands in the ammonium ferrous sulfate group were deficient and fuzzy compared with the NaCl group, suggesting that the natural structures of the proteins were damaged by ammonium ferrous sulfate. The protein bands for the ascorbic acid, potassium iodide, and sodium hyposulfite groups showed no differences compared with the NaCl group, indicating that the natural structures of the proteins were not destroyed by ascorbic acid, potassium iodide, or sodium hyposulfite. In SDS-PAGE, the gel and buffer contain a strong reductive material (SDS). The proteins of the samples are heated and denatured and separated according to the molecular weights of the protein subunits [16, 20]. The results showed that the protein bands in the ammonium ferrous sulfate group were deficient and fuzzy compared with the NaCl group, suggesting that the subunit structures of the proteins were damaged by ammonium ferrous sulfate. The protein bands of the ascorbic acid, potassium iodide, and sodium hyposulfite groups were the same as those of the NaCl group, indicating that ascorbic acid, potassium iodide, and sodium hyposulfite did not destroy the subunit structures of the proteins.

Thirdly, the effects of the reducing agents on Taq enzyme activity and DNA structure were investigated with qPCR. Comparing the experimental group to the control group, the pH value, ionic strength, and temperature were all consistent, so the experimental results were mainly related to the reducing agent. In the same reaction system, the experimental group of the same gene was compared with that of the control group. The increase in the Ct value indicated that amplification was inhibited, suggesting that the reducing agent had an effect on the enzyme or DNA. The results showed that ammonium ferrous sulfate had an inhibitory effect on the activity of enzymes and DNA in the range of 0.0390625 to 5 mmol/L. ascorbic acid, potassium iodide, and sodium hyposulfite had no inhibitory effect on the activity of enzymes and DNA in the concentration range used in the experiment.

The foregoing results showed that potassium iodide and sodium hyposulfite had no obvious inhibitory effects on the activities of the antibody or enzymes or DNA and protein structure. Potassium iodide and sodium hyposulfite were selected for the reversion test of oxidative damage. The experimental concentration was the maximum concentration that had no effect on the antibody in a previous experiment (50 mmol/L sodium hyposulfite and 50 mmol/L potassium iodide).

The final experiment was the possible repair of oxidative damage. The oxidative damage model was prepared using potassium permanganate. The antibody was damaged by the oxidant and then treated with reductant, and the antibody activity (OD values) was measured with ELISA. This experiment was divided into four groups: A, B, C, and D, and the OD values of the four groups were compared. The damage caused by K_2_MnO_4_ to the antibody was analyzed by comparing groups B and D [17]. The results showed that the OD values in group B were significantly lower than those in D group, indicating that the antibody activity was obviously damaged by potassium hypermanganate. Group C was compared with group D to investigate the effect of reducing agent on antibody activity. The results showed that the OD values in group C were not significantly different to those in group D. This suggested that the reducing agent had no significant effect on the activity of antibody. Group A was compared with group B to investigate whether the reducing agent could repair oxidative damage. The results showed that the OD values in group A were significantly higher than those in group B. This suggested that the reducing agent had a repairing effect on antibody damage caused by the oxidant. It is found that the reducing agent had only a partial repair effect on this damage by comparing OD values.

We comprehensively evaluated the effects of several reducing agents on the activity of biological macromolecules. These findings have some implications for the benefits and disadvantages of antioxidant supplementation. We found that reduced antibody activity due to an oxidant can be partially repaired by a reductant. Further studies on the specific mechanisms involved in oxidative stress and injury repair would be beneficial.

## Figures and Tables

**Table 1:** Inhibitory rates of different concentrations of ammonium ferrous sulfate and ascorbic acid on antibodies (%).

Concentration of reducing agents /(mmol/L)	ammonium ferrous sulfate	ascorbic acid
5	51.04±0.80^*∗∗∗*^	31.12±5.00^*∗∗∗*^
2.5	48.62±1.85^*∗∗∗*^	21.97±2.65^*∗∗∗*^
1.25	35.83±0.96^*∗∗∗*^	11.75±2.97^*∗∗*^
0.625	10.59±2.95^*∗∗*^	8.43±1.04^*∗*^
0.3125	6.50±2.32^*∗*^	3.02±1.78
0.15625	2.67±2.97	-1.36±2.97
0.078125	-0.94±2.10	0.95±2.02
0.0390625	1.38±0.70	2.48±1.54

Statistical significance was determined using the Student's *t*-test and is presented as ^*∗*^(*p* < 0.05), ^*∗∗*^(*p* < 0.01), or ^*∗∗∗*^(*p* < 0.001).

**Table 2:** Inhibitory rates of different concentrations of potassium iodide and sodium hyposulfite on antibodies (%).

Concentration of reducing agents / (mmol/L)	potassium iodide	sodium hyposulfite
50	0.45 ± 2.22	0.72 ± 2.08
25	4.98 ± 4.26	−0.39 ± 1.79
12.5	3.04 ± 2.06	−1.36 ± 3.32
6.25	1.25 ± 0.40	0.90 ± 3.15
3.125	4.10 ± 1.93	0.74 ± 0.31
1.5625	3.28 ± 2.04	1.57 ± 0.83
0.78125	−2.02 ± 0.82	0.49 ± 1.15
0.390625	1.87 ± 0.86	−0.02 ± 1.26

**Table 3:** Inhibitory rates of different concentrations of ammonium ferrous sulfate and ascorbic acid on Taq enzyme (%).

Concentration of reducing agents/(mmol/L)	ammonium ferrous sulfate		ascorbic acid	
	ß-actin	CRP	ß-actin	CRP
5	67.63±1.10^*∗∗∗*^	75.77±0.80^*∗∗∗*^	-0.06±0.14	-0.88±2.21
2.5	64.09±1.73^*∗∗∗*^	72.59±2.11^*∗∗∗*^	0.60±1.36	2.10±2.26
1.25	64.42±3.44^*∗∗∗*^	70.41±0.39^*∗∗∗*^	3.82±1.24	-0.06±4.16
0.625	15.26±3.01^*∗∗*^	13.59±2.65^*∗*^	0.36±4.04	-1.14±2.8
0.3125	3.96±0.58	7.15±4.32	2.41±0.75	-2.12±4.75
0.15625	3.33±2.01	5.43±0.82	2.71±6.75	-0.80±1.84
0.078125	0.03±2.50	3.19±2.13	-2.17±4.21	-2.45±2.16
0.0390625	0.17±1.52	4.25±1.50	-2.25±4.85	3.01±5.27

Statistical significance was determined using the Student's *t*-test and is presented as ^*∗*^(*p* < 0.05), ^*∗∗*^(*p* < 0.01), or ^*∗∗∗*^(*p* < 0.001).

**Table 4:** Inhibitory rates of different concentrations of potassium iodide and sodium hyposulfite on Taq enzyme (%).

Concentration of reducing agents/(mmol/L)	potassium iodide		sodium hyposulfite	
	ß-actin	CRP	ß-actin	CRP
50	3.97 ± 2.13	2.14 ± 0.32	7.24 ± 3.27	2.88 ± 0.13
25	1.26 ± 2.36	3.88 ± 1.04	3.94 ± 1.69	3.65 ± 0.70
12.5	0.83 ± 1.18	−2.43 ± 0.54	4.08 ± 2.87	2.53 ± 2.14
6.25	−2.81 ± 1.26	4.34 ± 1.73	3.73 ± 2.11	3.31 ± 1.35
3.125	2.23 ± 0.41	1.53 ± 1.82	3.98 ± 3.54	0.03 ± 2.86
1.5625	−0.84 ± 3.25	−0.92 ± 1.46	3.54 ± 3.25	1.31 ± 3.69
0.78125	3.48 ± 1.57	3.85 ± 0.04	0.41 ± 1.35	2.01 ± 1.41
0.390625	3.60 ± 2.35	−2.10 ± 0.65	2.11 ± 1.43	1.20 ± 0.68

**Table 5:** Inhibitory rates of different concentrations of ammonium ferrous sulfate and ascorbic acid on DNA (%).

Concentration of reducing agents/(mmol/L)	ammonium ferrous sulfate		ascorbic acid	
	ß-actin	CRP	ß-actin	CRP
5	66.81 ± 0.42^*∗∗∗*^	72.37 ± 0.68^*∗∗∗*^	−0.73 ± 0.46	−0.17 ± 1.81
2.5	61.21 ± 5.26^*∗∗∗*^	70.63 ± 4.50^*∗∗∗*^	1.05 ± 3.98	−2.14 ± 2.74
1.25	57.94 ± 1.99^*∗∗∗*^	40.81 ± 3.34^*∗∗∗*^	0.45 ± 1.74	0.42 ± 0.96
0.625	39.52 ± 4.09^*∗∗∗*^	31.90 ± 4.63^*∗∗∗*^	−1.79 ± 1.11	−1.93 ± 2.15
0.3125	23.81 ± 2.30^*∗∗∗*^	26.02 ± 1.85^*∗∗∗*^	0.48 ± 0.33	1.53 ± 2.26
0.15625	21.90 ± 0.50^*∗∗*^	23.82 ± 1.26^*∗∗*^	−0.85 ± 2.12	−0.48 ± 1.37
0.078125	17.80 ± 2.51^*∗∗*^	21.74 ± 4.66^*∗∗*^	−1.45 ± 0.76	2.13 ± 1.24
0.0390625	15.76 ± 4.82^*∗*^	19.42 ± 3.41^*∗∗*^	0.56 ± 0.43	0.71 ± 1.52

Statistical significance was determined using the Student's *t*-test and is presented as ^*∗*^(*p* < 0.05), ^*∗∗*^(*p* < 0.01), or ^*∗∗∗*^(*p* < 0.001).

**Table 6:** Inhibitory rates of different concentrations of potassium iodide and sodium hyposulfite on DNA (%).

Concentration of reducing agents/(mmol/L)	potassium iodide		sodium hyposulfite	
	ß-actin	CRP	ß-actin	CRP
50	1.70 ± 0.43	−1.84 ± 0.72	3.24 ± 1.03	3.59 ± 1.29
25	0.39 ± 0.67	0.15 ± 1.69	3.88 ± 0.42	3.47 ± 0.74
12.5	0.69 ± 1.38	0.57 ± 0.40	2.48 ± 0.82	1.88 ± 2.44
6.25	−1.58 ± 0.82	1.96 ± 1.35	4.10 ± 5.58	1.13 ± 1.38
3.125	3.2 ± 1.55	2.11 ± 1.89	3.80 ± 0.24	−0.23 ± 0.28
1.5625	2.86 ± 2.52	−1.33 ± 0.54	4.14 ± 4.69	4.01 ± 3.83
0.78125	1.14 ± 1.12	1.62 ± 1.82	−1.75 ± 1.48	−0.44 ± 1.59
0.390625	3.37 ± 2.44	2.21 ± 2.44	1.95 ± 1.50	2.20 ± 0.31

**Table 7:** The possible repair of HBsAb oxidative damage by potassium iodide.

Group	HBsAb	*p*
A	0.579 ± 0.037	0.0004
B	0.293 ± 0.016	

B	0.293 ± 0.016	0.0025
D	0.838 ± 0.137	

C	1.091 ± 0.142	0.0908
D	0.838 ± 0.137	

**Table 8:** The possible repair of HBsAb oxidative damage by sodium hyposulfite.

Group	HBsAb	*p*
A	0.641 ± 0.078	0.0021
B	0.323 ± 0.004	

B	0.323 ± 0.004	0.001
D	0.794 ± 0.027	

C	0.884 ± 0.099	0.2052
D	0.794 ± 0.027	

## Data Availability

The data used to support the findings of this study are included within the article.
